# A Polysaccharide Biosynthesis Locus in Vibrio parahaemolyticus Important for Biofilm Formation Has Homologs Widely Distributed in Aquatic Bacteria Mainly from *Gammaproteobacteria*

**DOI:** 10.1128/msystems.01226-21

**Published:** 2022-03-01

**Authors:** Ming Liu, Hailing Nie, Xuesong Luo, Shanshan Yang, Haozhe Chen, Peng Cai

**Affiliations:** a State Key Laboratory of Agricultural Microbiology, College of Resources of Environment, Huazhong Agricultural University, Wuhan, China; b College of Oceanography, Hohai University, Nanjing, China; University of California, Riverside

**Keywords:** *Vibrio parahaemolyticus*, biofilms, polysaccharide biosynthesis locus

## Abstract

Vibrio parahaemolyticus is a seafood-borne pathogen that poses a great threat to public health worldwide. It is found in either a planktonic cell or a biofilm form in the natural environment. The *cps* locus has been the only extensively studied polysaccharide biosynthesis gene cluster involved in biofilm formation for this bacterium. In this study, we found that an additional polysaccharide biosynthesis locus, *scv*, is also necessary for biofilm maturation. The *scv* locus is composed of two operons, and a loss of their expression leads to a defective biofilm phenotype. The transcription of the *scv* locus is under the control of a sigma 54-dependent response regulator, ScvE. In contrast, the quorum-sensing regulator AphA stimulates the expression of the *cps* locus and the *scvABCD* operon found in the *scv* locus. Bioinformatic analyses demonstrated that *scv* loci are divergent and widely distributed among 28 genera, including 26 belonging to the *Gammaproteobacteria* and 2 within the *Alphaproteobacteria*. We also determined that all *scv* locus-positive species are water-dwelling. Some strains of *Aeromonas*, Aliivibrio salmonicida, Pseudomonas anguilliseptica, Vibrio breoganii, and Vibrio scophthalmi probably acquired *scv* loci through insertion sequences and/or integrase-mediated horizontal gene transfer. Gene duplication and fusion were also detected in some *scv* homologs. Together, our results suggest that the genome of V. parahaemolyticus harbors two distinct polysaccharide biosynthesis loci, which may play a role in fine-tuning biofilm development, and that *scv* loci likely evolved by horizontal gene transfer, gene loss, gene duplication, and fragment fusion.

**IMPORTANCE** Polysaccharides are the major component of biofilms, which provide survival advantages for bacteria in aquatic environments. The seafood-borne pathogen V. parahaemolyticus possesses a functionally uncharacterized polysaccharide biosynthesis locus, *scv*. We demonstrated that the *scv* locus is important for biofilm maturation and that *scv* expression is positively regulated by ScvE. Strains from 148 aquatic bacterial species possess *scv* homolog loci. These bacterial species belong to 28 genera, most of which belong to the *Gammaproteobacteria* class. The evolution and diversification of *scv* loci are likely driven by horizontal gene transfer, gene loss, gene duplication, and fragment fusion. Our results provide new insights into the function and evolution of this widespread polysaccharide biosynthesis locus.

## INTRODUCTION

Vibrios are biofilm-forming bacteria that are widespread in aquatic environments. Biofilm encapsulation protects *Vibrio* species from predators and enhances their resistance to harsh environmental conditions, including pH shifts, antimicrobials, and oxidative stress. Conversely, *Vibrio* in the planktonic cell form is often recovered from seawater, indicating a flexible lifestyle (1–3). In addition to being the primary biofilm component, polysaccharides are beneficial for the survival and adaptation of *Vibrio* species. For example, polysaccharides enable Vibrio cholerae to resist attack by the type VI secretion system (T6SS) and are vital for intestinal colonization (4, 5).

Vibrio parahaemolyticus is often reported to infect human beings through seafood consumption. V. parahaemolyticus is persistent during seafood processing and storage, likely due to the biofilms that it forms (6, 7). Therefore, the mechanism of V. parahaemolyticus biofilm formation has attracted considerable interest in recent decades. Chromosome II of V. parahaemolyticus pandemic strain RIMD2210633 harbors a polysaccharide synthesis gene cluster, *cps*, which is essential for producing biofilms and is regulated by quorum sensing (QS) (8). At a low cell density, ScrA produces a small amount of an unknown compound, which binds to ScrB. The compound-ScrB complex then activates ScrC to synthesize cyclic di-GMP (c-di-GMP). CpsQ, a downstream c-di-GMP-activated regulator, stimulates the expression of the *cps* locus (8). The master QS regulator AphA is also expressed at a low cell density and indirectly stimulates the production of c-di-GMP (9). At a high cell density, a master QS regulator, OpaR, is expressed. The loss of *aphA* but not *opaR* renders the bacteria less virulent and reduces biofilm formation after 24 h of growth (10, 11). ScrO, a CpsQ homolog regulator, and H-NS can also activate the expression of *cpsA* (12, 13).

V. parahaemolyticus possesses a second polysaccharide biosynthesis gene cluster, which shows low homology to the *syp* locus in Aliivibrio fischeri (14). However, the function and regulation of this second polysaccharide biosynthesis gene cluster during biofilm development are unclear. In Aliivibrio fischeri, the *syp* locus consists of 18 genes. It is essential for both biofilm formation and colonization of squid. *syp* locus transcription is likely directly activated by phosphorylated SypG, which is a sigma 54-dependent response regulator (14, 15). Further studies show that SypF, RscS, and HahK are the activators of SypG (16–18). The *syp* locus could also be homologous to the *rbd* locus in Vibrio vulnificus (19). A functional *syp*-like locus was also detected in Vibrio diabolicus (20). Therefore, we hypothesize that the second polysaccharide biosynthesis gene cluster in V. parahaemolyticus, a homolog of the *syp* locus, now referred to as *scv* (*syp*-like locus in V. parahaemolyticus), could be important for biofilm formation. The transcription of the *scv* locus could be regulated by ScvE, the ortholog of SypG. To validate this hypothesis, we constructed genetically modified strains to study their biofilm development, polysaccharide biosynthesis, and gene expression. We also analyzed *scv*-homologous loci *in silico* to probe the probable mechanism for the evolution of this polysaccharide biosynthesis gene cluster.

## RESULTS AND DISCUSSION

### The *scv* locus is essential for biofilm formation.

**FIG 1:**
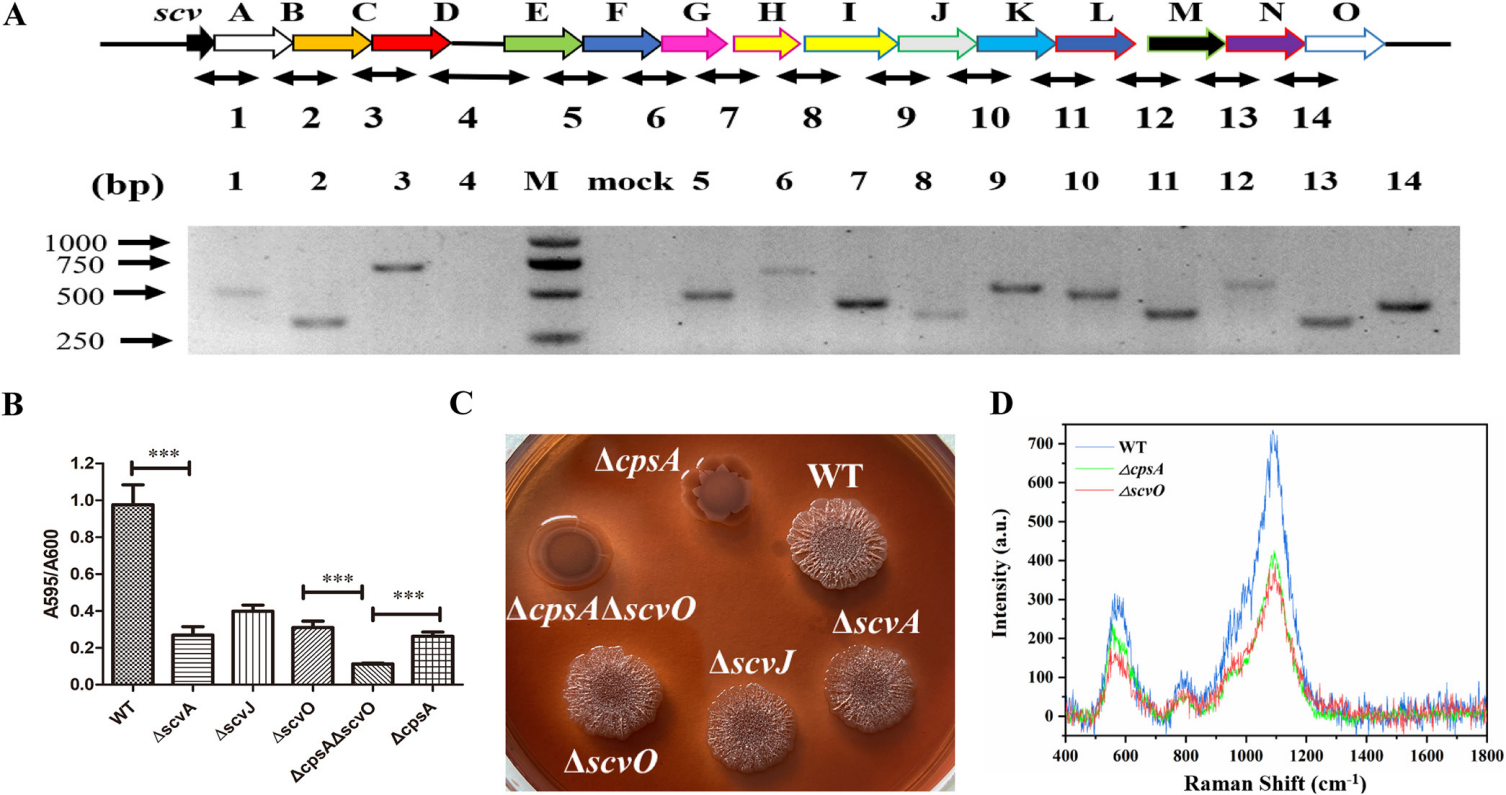
The *scv* locus is important for biofilm formation by the production of polysaccharide. (A) Schematic representation of the *scv* locus. Each gene in the *scv* cluster is marked with a different-colored open arrow, and the regions amplified for RT-PCR analysis are indicated below. One hundred nanograms of cDNA was used as the template for each reaction. For the mock reaction, no reverse transcriptase was added and was used as a control against genomic contamination of RNA preparations. (B) Biofilm formation of the WT, Δ*scvA*, Δ*scvJ*, Δ*scvO*, Δ*cpsA*, and Δ*scvO* Δ*cpsA* strains after 24 h of incubation at 30°C. The biofilm was stained with crystal violet. (C) Colony morphologies of the WT, Δ*scvA*, Δ*scvJ*, Δ*scvO*, Δ*cpsA*, and Δ*scvO* Δ*cpsA* strains on Congo red plates. (D) SERS analysis of WT, Δ*scvO*, and Δ*cpsA* biofilms. a.u., arbitrary units. Statistical comparisons were performed by a *t* test. ***, *P* < 0.001.

Homolog genes associated with the *syp* locus were observed in V. parahaemolyticus in 2005, and the deletion of the ortholog of *sypQ* (referred to here as *scvN*) resulted in a biofilm formation defect (14, 21). However, both the function of the *scv* gene cluster in biofilm development and the regulatory mechanism for *scv* locus transcription are largely unknown. Through bioinformatic analysis, we found that in the genome of V. parahaemolyticus pandemic strain RIMD2210633, the *scv* locus consists of 15 genes located on chromosome I, which show 33% to 67% homology to their corresponding orthologs. No orthologs of SypE-, SypF-, or SypM-encoding genes were observed in this gene cluster (see Table S1 in the supplemental material). To further characterize the locus, we asked if the *scv* locus genes are organized into operons in strain RIMD2210633. Reverse transcription-PCR (RT-PCR) showed that *scvA*, *scvB*, *scvC*, and *scvD* are in one operon, while the other *scv* genes are in a different operon (Fig. 1A). These results indicate that the genetic organization of the *scv* locus differs from those of *syp* and *rbd*, consisting of 18 and 17 genes, respectively, both organized into four operons (14, 20). Furthermore, the function and regulation of gene expression for the *syp*, *rbd*, and *scv* loci could differ. For example, unlike the transcription levels of the *syp* and *rbd* loci, which were very low (14, 19), the expression of the *scv* locus was easily detectable under laboratory conditions (Fig. 1A).

10.1128/mSystems.01226-21.3 TABLE S1 Putative functions of Scv proteins. Download Table S1, DOCX file, 0.02 MB. Copyright © 2022 Liu et al. 2022 Liu et al. https://creativecommons.org/licenses/by/4.0/ This content is distributed under the terms of the Creative Commons Attribution 4.0 International license.

Next, to determine if the *scv* locus was involved in biofilm formation, we constructed Δ*scvA*, Δ*scvJ*, and Δ*scvO* strains and compared their biofilms to those of the Δ*cpsA* and wild-type (WT) strains. The results showed that although the Δ*scvA*, Δ*scvJ*, Δ*scvO*, and Δ*cpsA* strains produced similar amounts of biofilm, they all produced about 60% less biofilm than the WT strain. Further incubations of the WT and Δ*scvO* strains did not significantly increase biofilms (Fig. S1). In addition, the deletion of *scvO* in the Δ*cpsA* strain further decreased biofilm formation by approximately 50% compared with the Δ*scvO* and Δ*cpsA* strains (Fig. 1B). WT cells formed rugose and opaque colonies on Congo red agar, while colonies of the Δ*cpsA* strain were smooth and cloudy, and those of the Δ*cpsA* Δ*scvO* strain appeared transparent. The morphologies of Δ*scvA*, Δ*scvJ*, and Δ*scvO* cells developed on Congo red agar were similar but differed from those of Δ*cpsA*, Δ*cpsA* Δ*scvO*, and WT cells (Fig. 1C). These results indicate that the *cps* locus and the *scv* locus are two primary loci essential for biofilm formation in V. parahaemolyticus and that these loci have distinct roles in colony formation. As the biofilm production and morphologies of the Δ*scvA*, Δ*scvJ*, and Δ*scvO* strains were similar, while the biofilm production of the Δ*cpsA* Δ*scvO* strain was nearly completely lost (∼90% less than that of the WT), we selected the Δ*scvO* strain as the representative *scv* knockout mutant in the following assays.

10.1128/mSystems.01226-21.1 FIG S1 Impact of different incubation times on V. parahaemolyticus biofilm formation. Biofilm formation of the WT and Δ*scvO* strains at 30°C was assessed after 24 h and 48 h of incubation, respectively. The biofilm was stained with crystal violet. Statistical comparisons were performed by a *t* test. ns, not significant (*P > *0.05). Download FIG S1, TIF file, 0.2 MB. Copyright © 2022 Liu et al. 2022 Liu et al. https://creativecommons.org/licenses/by/4.0/ This content is distributed under the terms of the Creative Commons Attribution 4.0 International license.

### The *scv* locus is required for the production of polysaccharide.

To determine whether defective biofilm formation is due to the reduction in polysaccharide production in mutants, real-time surface-enhanced Raman spectroscopy (SERS) was used to quantify the chemical characteristics of WT, Δ*scvO*, and Δ*cpsA* biofilms. Our results showed that the intensities ranged from 1,050 to 1,150 cm^−1^ for the Δ*scvO* and Δ*cpsA* strains, while WT intensities of 560 to 620 cm^−1^ were the highest. Intensities from 1,050 to 1,150 cm^−1^ in the Δ*scvO* and Δ*cpsA* strains were similar, while a slightly higher band between 560 and 620 cm^−1^ was detected in the Δ*cpsA* strain (Fig. 1D). Because peaks of Raman spectra in the ranges of 560 to 582 cm^−1^ and 1,090 to 1,095 cm^−1^ represent C-O-C glycosidic rings from polysaccharides (22), these results indicated that similar to the *cps* locus, the *scv* locus is also involved in the biosynthesis of polysaccharide. Because Raman spectra cannot differentiate the detailed chemical compositions of polysaccharides synthesized by the *scv* and *cps* loci, further studies are needed to characterize the biochemical properties of the polysaccharides synthesized by these loci. Furthermore, given that (i) the loss of *scvN* caused a complete inability to synthesize poly-*N*-acetylglucosamine in V. parahaemolyticus (21), (ii) many bacteria produce this chemical at low levels (23), and (iii) a Raman band at 1,445 cm^−1^ represents poly-*N-*acetylglucosamine, which we and other researchers did not detect in biofilms of V. parahaemolyticus (22, 24), we concluded that poly-*N*-acetylglucosamine is likely a very small fraction of the polysaccharide synthesized by the *scv* locus, while the *cps* locus is not involved in producing poly-*N*-acetylglucosamine. Therefore, it is likely that the *scv* and *cps* loci synthesize structurally different polysaccharides.

### The *scv* locus contributes to mature biofilm formation.

**FIG 2:**
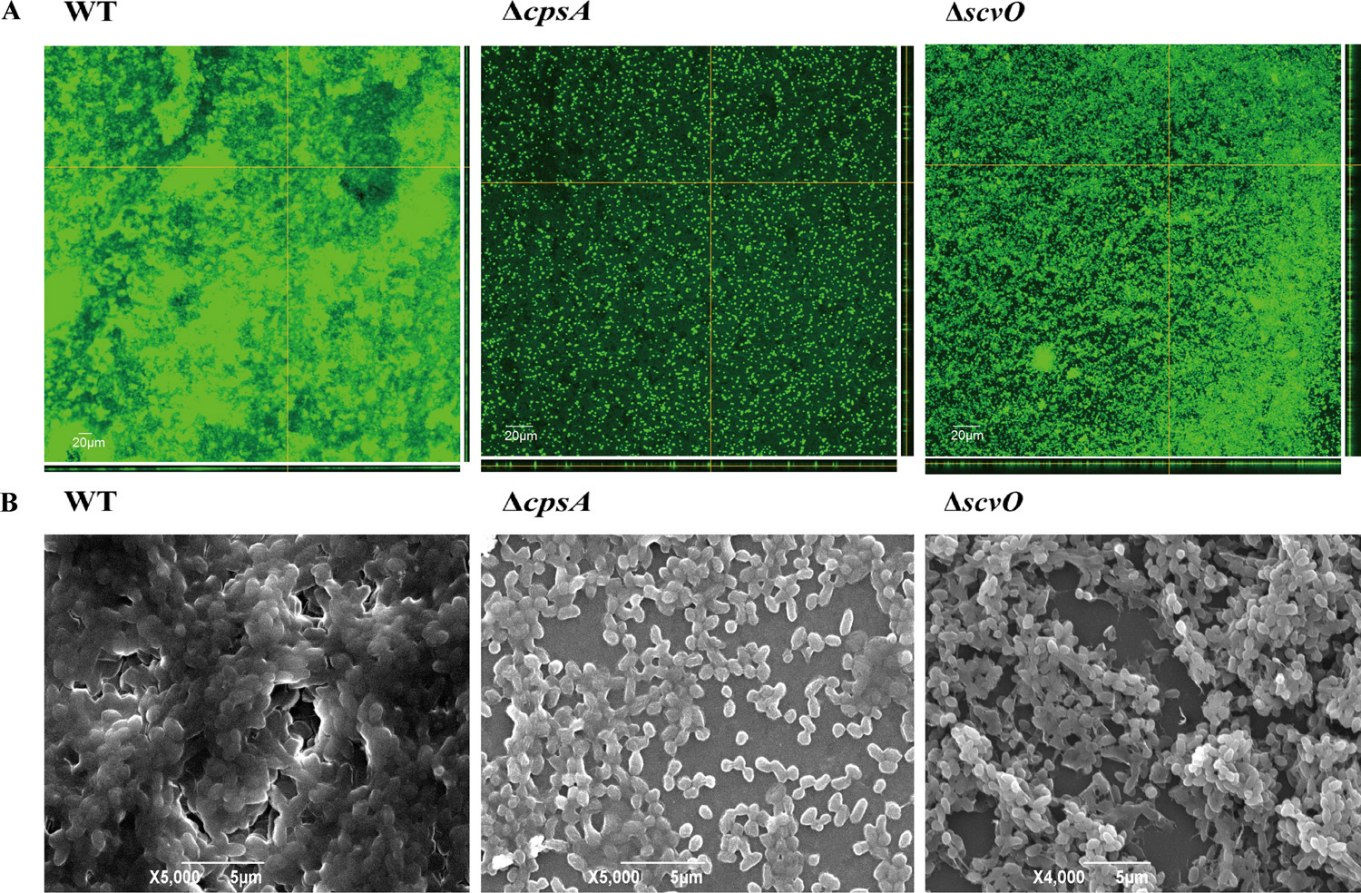
SEM and CLSM images of biofilms formed by V. parahaemolyticus strains. (A) CLSM images of WT, Δ*scvO*, and Δ*cpsA* biofilms. Cells were stained with SYTO-9. (B) SEM images of WT, Δ*scvO*, and Δ*cpsA* biofilms.

Having established that the *scv* locus is essential for biofilm formation, we questioned if the contribution of the *scv* locus to biofilm composition is distinct from that of the *cps* locus. We used confocal laser scanning microscopy (CLSM) and scanning electron microscopy (SEM) to compare the Δ*scvO* biofilm with those of the Δ*cpsA* and WT strains. The results showed that the WT strain produced thick, densely packed, robust biofilms, while biofilms of the Δ*scvO* and Δ*cpsA* strains were thin and nonmature. In the Δ*cpsA* biofilm, many single cells were observed, while in the Δ*scvO* biofilm, cells formed clumps, which were only loosely bound together (Fig. 2A and B). These results indicated that both the *cps* and *scv* loci are required for mature biofilm formation and have different roles in forming the biofilm structure.

### ScvE positively regulates *scv* locus transcription.

**FIG 3:**
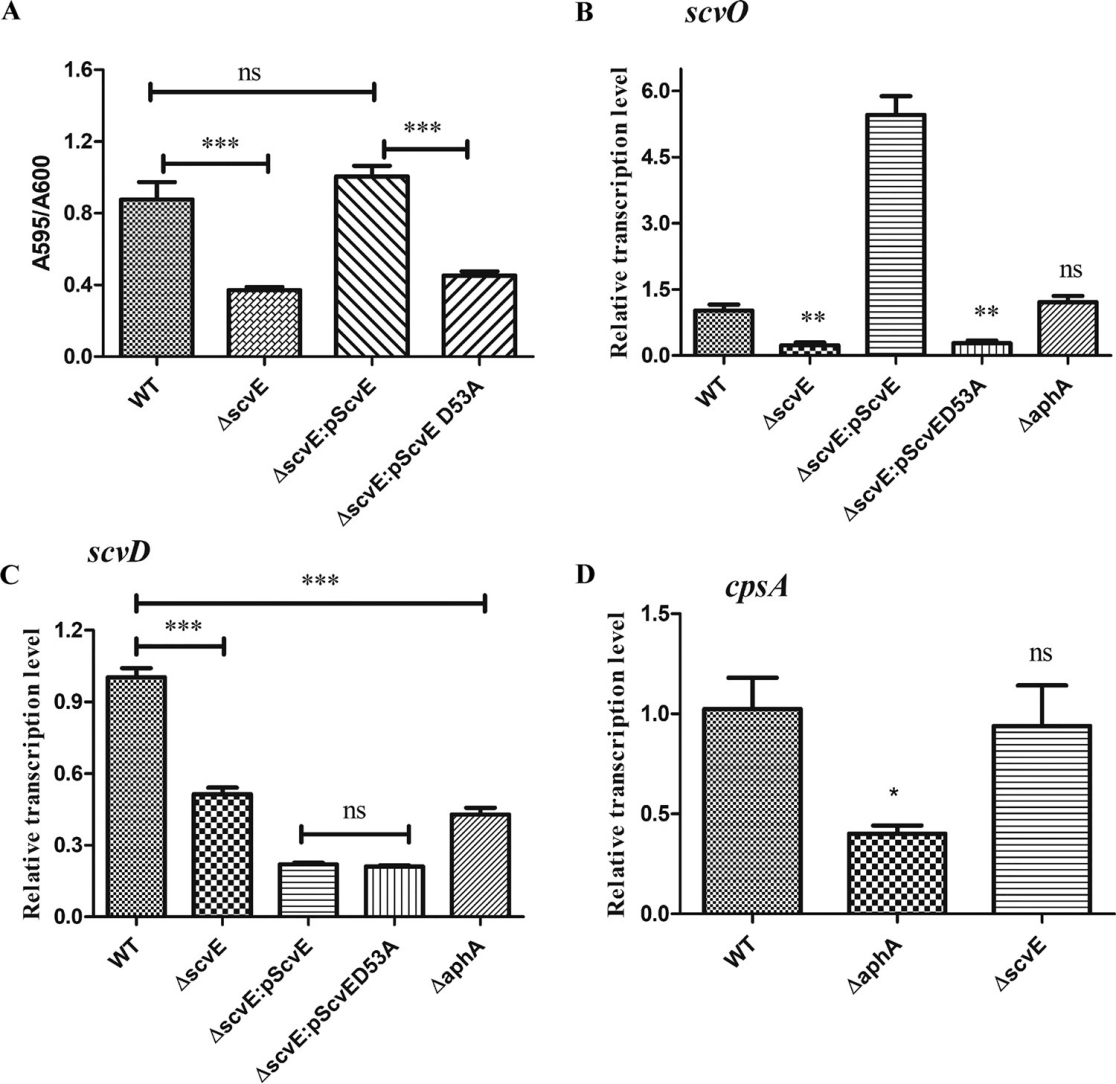
ScvE stimulates the expression of the *scv* locus. (A) Biofilm formation of the WT, Δ*scvE*, Δ*scvE*::pScvE, and Δ*scvE*::pScvE^D53A^ strains after 24 h of incubation at 30°C. The biofilm was stained with crystal violet. (B) ScvE controls the expression of *scvO*. (C) ScvE and AphA stimulate the transcription of *scvD*. (D) AphA positively regulates the transcription of *cpsA*. Statistical comparisons were performed by a *t* test. *, *P < *0.05; **, *P* < 0.01; ***, *P* < 0.001; ns, not significant (*P > *0.05).

Given that (i) SypG and RbdG are functional (14, 19) and (ii) SypG, RbdG, and ScvE are homologous proteins, we hypothesized that ScvE regulates biofilm development. First, we constructed the Δ*scvE* strain. Biofilm formation assays showed that the WT strain produced about 2-fold more biofilm than the Δ*scvE* strain. The complementary Δ*scvE*::pScvE strain produced a level of biofilm similar to that of the WT (Fig. 3A). As ScvE possesses the receiver (REC) domain, to test whether the conserved aspartate residue in the REC domain (Asp53) has any role in biofilm formation, the Δ*scvE*::pScvE^D53A^ strain was generated. We found that its biofilm formation ability was similar to that of the Δ*scvE* strain (Fig. 3A). Real-time quantitative reverse transcription-PCR (qRT-PCR) results showed that in the Δ*scvE* strain, the transcription levels of *scvO* and *scvD* dropped about 70% and 60%, respectively, compared with those of the WT. However, *scvE* did not significantly affect *cpsA* expression; instead, *aphA* regulated the transcription of both *cpsA* and *scvD* and not *scvO*. Moreover, in the Δ*scvE*::pScvE strain, *scvO* expression was upregulated, while the expression level of *scvD* remained as low as that in the Δ*scvE*::pScvE^D53A^ strain (Fig. 3B to D). The failure of the Δ*scvE*::pScvE strain to stimulate the transcription of *scvD* may be due to the polar effect from the *scvABCD* operon since *scvD* is a downstream gene in this operon and could be less affected by *scvA* promoter-dependent regulation. These results indicate that *scvE* positively regulates the transcription of the *scvABCD* and *scvEFGHIJKLMNO* operons, while the QS regulator AphA modulates the expression of the *cps* locus and the *scvABCD* operon.

### The *scv* locus is insensitive to low temperatures and calcium levels.

**FIG 4:**
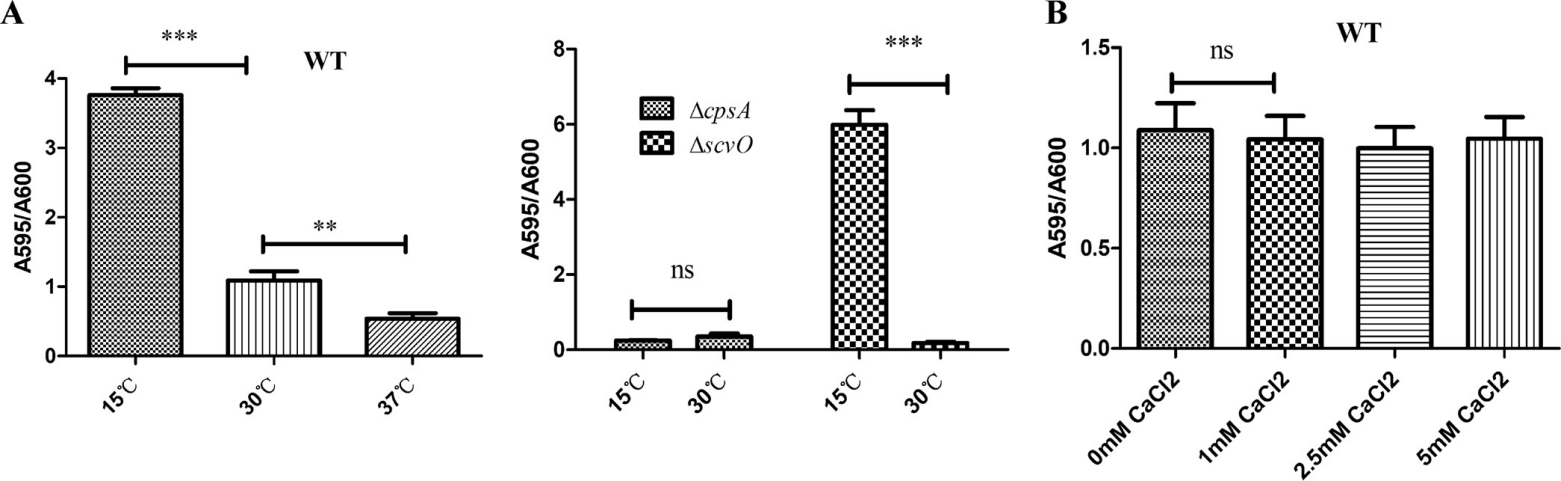
Impact of temperature and calcium on V. parahaemolyticus biofilm formation. (A) Biofilm formation of the WT, Δ*scvO*, and Δ*cpsA* strains at different temperatures after 24 h of incubation. (B) Biofilm formation of the WT under different calcium levels in HIS medium after 24 h of incubation at 30°C. The biofilm was stained with crystal violet. Statistical comparisons were performed by a *t* test. **, *P* < 0.01; ***, *P* < 0.001; ns, not significant (*P > *0.05).

Considering that low temperatures and high calcium levels can promote biofilm formation in V. cholerae, which increases its adaptivity in response to environmental stresses (25, 26), we analyzed the role of the *cps* and *scv* loci in biofilm development under different temperatures and calcium concentrations. We found that low temperatures enhanced the biofilm formation of the WT and Δ*scvO* strains but not that of the Δ*cpsA* strain (Fig. 4A). Various calcium levels did not affect the biofilm formation of the WT (Fig. 4B). Likely, the *scv* locus is not responsive to low temperature or calcium, whereas low temperature stimulates biofilm formation via the *cps* locus in V. parahaemolyticus. Finding out which environmental signals trigger the expression of the *scv* locus will shed light on the mechanisms by which V. parahaemolyticus fine-tunes biofilm development.

### *scv* loci are divergent and widespread among 28 genera.

**FIG 5:**
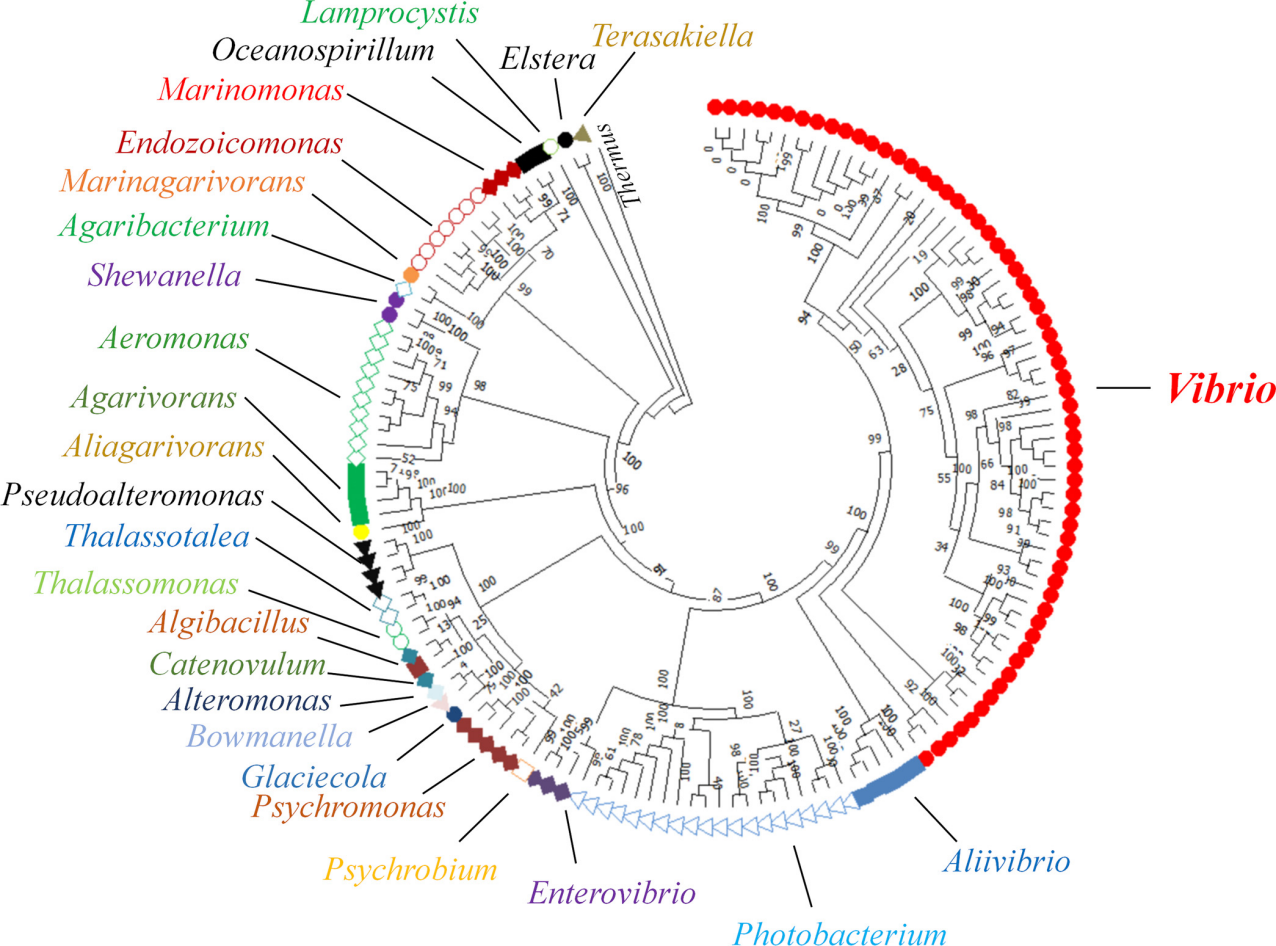
Distribution of *scv* loci in diverse species. The maximum likelihood phylogenetic tree was built using *rpoB* sequences (MEGA X). Numbers at the nodes are percent bootstrap values based on 500 replicates. *Thermus* was used as an outgroup. Sequences from the same genus are marked with the same color. Each symbol represents one bacterial species. Elstera litoralis Dia-1, Enterovibrio coralii CAIM 912, Pseudomonas anguilliseptica DSM 12111, and *Thalassolituus* sp. HI0120 were not included in the phylogenetic analysis since their complete *rpoB* sequences are not available.

**FIG 6:**
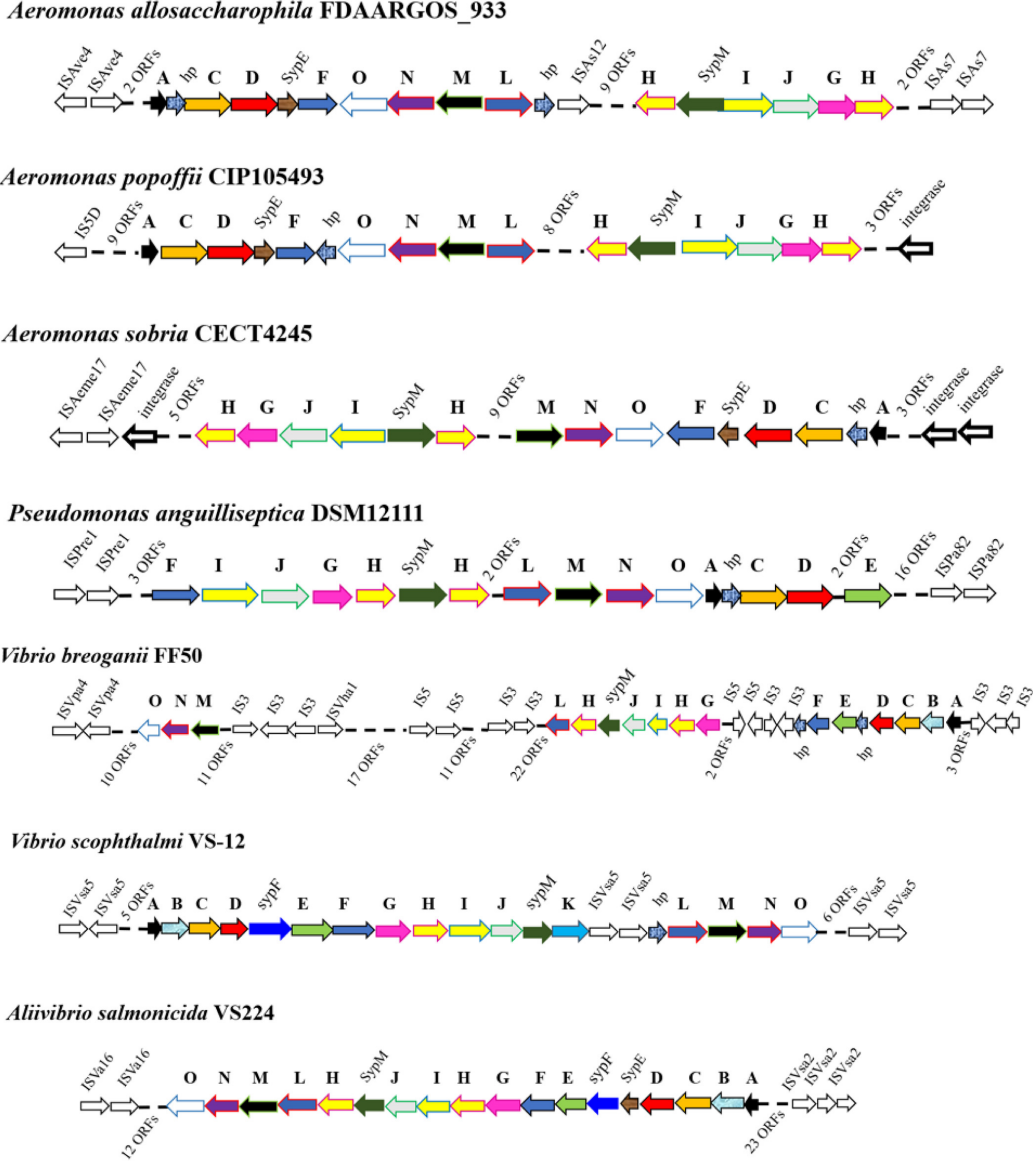
An insertion sequence and/or integrase is detectable upstream and downstream of the *scv* loci in the same bacterial species. Each ortholog gene in the *scv* locus is marked with an arrow of a different color. The same-colored arrows indicate orthologous genes. Insertion sequences were identified using IS Finder. ORFs, open reading frames.

Given that *scv* loci possessed by two taxa, V. parahaemolyticus and Aliivibrio fischeri, are functional, we investigated the broader prevalence of the *scv* loci. We utilized the sequences for ScvC, ScvL, and ScvO as queries in PSI-BLAST. The results showed that in addition to *Vibrio*, several other genera also contain orthologs of ScvC, ScvL, and/or ScvO, including *Aeromonas*, *Agaribacterium*, *Agarivorans*, *Aliivibrio*, *Alteromonas*, *Elstera*, *Endozoicomonas*, *Enterovibrio*, and *Marinagarivorans*. Next, the genomes of all the bacterial species with orthologs were downloaded from the NCBI database and reannotated using RAST. *scv* orthologs were detected in the genomes of 148 bacterial species, which are affiliated with 28 genera in eight orders, including *Aeromonadales*, *Alteromonadales*, *Cellvibrionales*, *Chromatiales*, *Oceanospirillales*, *Pseudomonadales*, *Rhodospirillales*, and *Vibrionales*. All other genera belong to the *Gammaproteobacteria*, except for *Elstera* and *Terasakiella*, which are members of the *Alphaproteobacteria* (Fig. 5). All *scv*-positive strains are water-dwelling, and the majority are *Vibrio* species (62 species). Given that the *syp* locus is important for Aliivibrio fischeri to colonize squid, its natural host, and that *scvN* is indispensable for V. parahaemolyticus to colonize fish (14, 21), *scv* locus-positive bacterial species likely express these loci to colonize aquatic hosts. In addition, it is noteworthy that only a few species possess the *scv* locus in some genera, such as Shewanella corallii, *Shewanella* sp. SNU WT4, Oceanospirillum multiglobuliferum, Oceanospirillum sanctuarii, and Pseudomonas anguilliseptica. In the genome sequences of Aeromonas allosaccharophila strain FDAARGOS_933, Aeromonas popoffii strain CIP105493, Aeromonas sobria strain CECT4245, Aliivibrio salmonicida strain VS224, Pseudomonas anguilliseptica strain DSM12111, Vibrio breoganii strain FF50, and Vibrio scophthalmi strain VS-12, insertion sequence elements and/or integrases are detectable upstream and downstream of the *scv* loci, indicating that these strains acquired *scv* gene clusters by insertion sequences and/or integrase-mediated horizontal gene transfer (Fig. 6).

**FIG 7:**
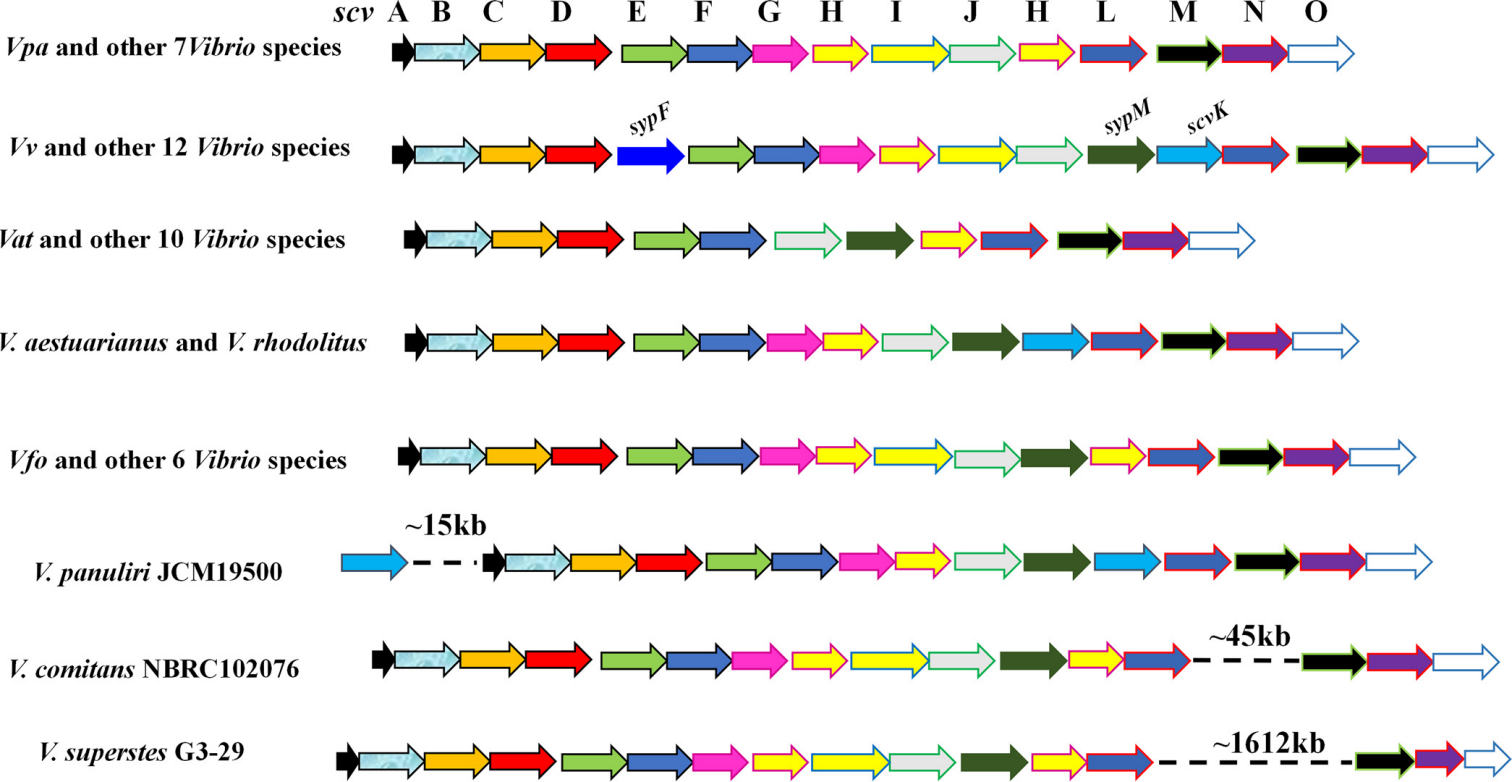
Genetic structures of the *scv* loci among *Vibrio* species. Each orthologous gene in the *scv* locus is marked with an arrow of a different color. The same-colored arrows indicate orthologous genes. Abbreviations of species names: *Vpa*, V. parahaemolyticus; *Vv*, V. vulnificus; *Vat*, *V. atlanticus*; *Vfo*, *V. fortis*.

Previous studies suggested that surface polysaccharide synthesis loci are hot spots shaped by selection forces in *Enterobacteriales* and some pathogenic bacteria. These selection forces can drive the structural diversification of polysaccharide biosynthesis gene clusters (27, 28). Consistent with this viewpoint, we found that *scv* loci are highly divergent. For instance, all *scv* locus-positive *Aeromonas* and *Endozoicomonas* species do not possess an ortholog of *scvB*, a putative OmpA-encoding gene, while all *scv* locus-positive *Marinomonas*, *Photobacterium*, and *Psychromonas* species do (see Table S4 in the supplemental material). Structural differences in the *scv* gene clusters are also frequently detected among species of the same genus. For example, the orthologs of *sypF* were not observed in the *scv* loci of 38 *Vibrio* species but were present in 24 other *scv* locus-positive *Vibrio* species. Fifty-three *Vibrio* species possess an ortholog of *sypM*, while 9 species, including V. parahaemolyticus, *V. diabolicus*, V. alginolyticus, *V. azureus*, *V. campbellii*, *V. hyugaensis*, *V. jasicida*, *V. owensii*, and *V. rotiferianus*, do not. Similarly, orthologs of *scvG* and/or *scvI* are absent from some *scv* locus-positive vibrios, while 11 other strains do not possess orthologs of *sypF*, *scvG*, *scvI*, or *scvK*. Interestingly, in *V. comitans* strain NBRC102076, *V. panuliri* strain JCM19500, and *V. superstes* strain G3-29, the physical distance among genes annotated as being members of the *scv* loci ranges from ∼15 kb to ∼1,612 kb (Fig. 7 and Table S5). This suggests that extensive fragment recombination may occur during the evolution of *scv* loci in these *Vibrio* species. In addition, it is worth noting that all *scv* locus-positive *Vibrio* species possess an ortholog of *scvN* (Table S5), and *scvN* is involved in poly-*N*-acetylglucosamine biosynthesis in V. parahaemolyticus (21). All *scv* locus-positive *Vibrio* species likely produce poly-*N*-acetylglucosamine as a component of the polysaccharides.

10.1128/mSystems.01226-21.6 TABLE S4 Twenty-seven genera of *scv* locus-positive bacterial species. A protein domain search was performed with SMART. p, positive; pp, two paralogs; ppp, three paralogs; pppp, four paralogs; f, different protein domains integrated into one molecule; neg., negative. Although Photobacterium sanguinicancri ME15, Elstera litoralis Dia-1, Photobacterium indicum ATCC 19614, Photobacterium leiognathi JS01, and *Thalassolituus* sp. HI0120 possessed orthologous genes of the *scv* locus, they were excluded from domain analysis because discontinued contigs may indicate low sequence quality. Download Table S4, XLSX file, 0.02 MB. Copyright © 2022 Liu et al. 2022 Liu et al. https://creativecommons.org/licenses/by/4.0/ This content is distributed under the terms of the Creative Commons Attribution 4.0 International license.

10.1128/mSystems.01226-21.7 TABLE S5 *scv* locus-positive *Vibrio* species. A protein domain search was performed with SMART. p, positive; pp, two homologs; neg., negative. Download Table S5, XLSX file, 0.01 MB. Copyright © 2022 Liu et al. 2022 Liu et al. https://creativecommons.org/licenses/by/4.0/ This content is distributed under the terms of the Creative Commons Attribution 4.0 International license.

The genetic differences in *scv* loci can also be caused by gene duplication and domain rearrangement. For instance, orthologs of *scvH* are duplicated in several species such as Thalassomonas viridans, Thalassomonas actiniarum, Shewanella corallii, *Shewanella* sp. SNU WT4, Pseudomonas anguilliseptica, and 29 *Vibrio* species. *Agarivorans* species have three *scvN* paralogs (Table S4). Moreover, in most *scv* locus-positive species, *sypM* encodes a single hexapeptide transferase domain, and *scvI* encodes a single polysaccharide biosynthesis domain. However, orthologs of *sypM* encode duplicated hexapeptide transferase domains in Pseudomonas anguilliseptica, Thalassomonas actiniarum, *Thalassotalea* sp. M1531, Aeromonas enteropelogenes, Aeromonas taiwanensis, Agaribacterium haliotis, Aliagarivorans marinus, *Bowmanella* sp. JS7-9, and 19 *Vibrio* species (Fig. S2A and Tables S4 and S5). Orthologs of *scvI* encode two polysaccharide biosynthesis domains in *Alteromonas sediminis*, Endozoicomonas elysicola, Endozoicomonas numazuensis, *Glaciecola* sp. HTCC2999, and *Thalassotalea* sp. M1531 (Fig. S2B). Intriguingly, orthologs of *scvN* and *scvO* likely fuse into one gene in some strains of *Alteromonas sediminis*, Marinagarivorans algicola, *Photobacterium*, *Agarivorans*, and *Pseudoalteromonas* (Fig. S2C and Table S4), while orthologs of *scvF* and *scvG* likely fuse into one gene in Agaribacterium haliotis (Fig. S2D).

10.1128/mSystems.01226-21.2 FIG S2 Domain architectures of Scv ortholog proteins. (A) Domains of SypM. (B) Domains of ScvI. (C) Ortholog of *scvN* and *scvO* likely fused into one gene. (D) Ortholog of *scvF* and *scvG* likely fused into one gene. A protein domain search was performed with SMART. In the upper lane, the domain does not rearrange. Download FIG S2, TIF file, 0.2 MB. Copyright © 2022 Liu et al. 2022 Liu et al. https://creativecommons.org/licenses/by/4.0/ This content is distributed under the terms of the Creative Commons Attribution 4.0 International license.

### Conclusions.

In summary, we revealed that the *scv* locus synthesizes the biofilm-related polysaccharide, which is structurally and functionally different from that synthesized by the *cps* locus. The polysaccharide synthesized by the *cps* locus dramatically affects multicellular aggregation, while the *scv* locus has a modest impact on biofilm formation. In addition, *scvE* can regulate the transcription of the *scvABCD* and *scvEFGHIJKLMNO* operons, while *aphA* stimulates the expression of only the first operon in the *scv* locus. Moreover, *scv* homolog loci are divergent and found in a large number of diverse aquatic bacterial species. Horizontal gene transfer, gene loss, gene duplication, and fragment fusion are the likely driving forces behind the evolution of *scv* loci. Our results provide new insights into the function and evolution of this widespread polysaccharide biosynthesis locus.

## MATERIALS AND METHODS

### Bacterial strains and growth conditions.

V. parahaemolyticus strains were cultured in heart infusion medium (Difco) supplemented with 0.5% sodium chloride (HIS medium) at 30°C unless otherwise mentioned, and Escherichia coli strains were cultured in Luria-Bertani (LB) medium at 37°C. Culture media were supplemented with chloramphenicol at final concentrations of 5 μg/mL for V. parahaemolyticus and 25 μg/mL for E. coli or 1 mM isopropyl β-d-thiogalactoside (IPTG) if necessary.

Congo red plates were prepared using HIS medium supplemented with 10 mL/L Congo red and 5 mL/L Coomassie blue G-250. Two microliters of the cultures grown overnight were spotted onto the Congo red plates and incubated at 30°C for 3 days. The pictures shown are from one of at least three independent experiments.

### Strain construction.

The knockout mutant was obtained using the pDM4 vector as previously described (29). Briefly, ∼800-bp upstream and downstream fragments of the target gene were amplified, and the first-round products were used as the templates in the second-round PCR. Next, the PCR product was inserted into the suicide vector pDM4. The recombinant was transformed into E. coli S17-1 λ*pir* and then conjugated with V. parahaemolyticus. The mutant was selected and confirmed by PCR. For obtaining the Δ*scvE*::pScvE strain, the entire coding region of *scvE* with an N-terminal ribosome binding site was amplified, inserted into pMMB207, and then transconjugated into the Δ*scvE* strain. Site-directed mutagenesis was performed using a GeneArt site-directed mutagenesis kit (Thermo) according to the manufacturer’s instructions. The primers used are listed in Table S3 in the supplemental material.

10.1128/mSystems.01226-21.4 TABLE S2 Strains and plasmid used in this study. Download Table S2, DOCX file, 0.02 MB. Copyright © 2022 Liu et al. 2022 Liu et al. https://creativecommons.org/licenses/by/4.0/ This content is distributed under the terms of the Creative Commons Attribution 4.0 International license.

10.1128/mSystems.01226-21.5 TABLE S3 Primers used in this study. Download Table S3, DOCX file, 0.02 MB. Copyright © 2022 Liu et al. 2022 Liu et al. https://creativecommons.org/licenses/by/4.0/ This content is distributed under the terms of the Creative Commons Attribution 4.0 International license.

### Microplate biofilm formation assay.

V. parahaemolyticus strains grown overnight were diluted 1:50 into HIS broth and incubated at 30°C at 200 rpm for 2 h, the culture was then adjusted to an optical density at 600 nm (OD_600_) of 0.05, and 200 μL bacterial cells was added to wells of 96-well microplates (8 wells per strain). Following incubation of the bacterium at 100 rpm for 24 h, the OD_600_ of each well was recorded using a microplate reader. After the decantation of the planktonic cells in each well, the wells were washed twice with phosphate-buffered saline (PBS) (137 mM NaCl, 2.7 mM KCl, 10 mM Na_2_HPO_4_, 1.8 mM KH_2_PO_4_), 220 μL crystal violet (0.1%, wt/vol) was then added to each well, and the wells were stained at room temperature for 20 min. The crystal violet was then decanted out, each well was washed 3 times with PBS, and 220 μL 100% ethanol was added. The OD_595_ of each well was measured using a microplate reader. The relative ability for biofilm formation was calculated based on the OD_595_/OD_600_. Three independent experiments were performed.

### CLSM, SEM, and SERS.

For CLSM analysis, V. parahaemolyticus strains grown overnight were diluted 1:50 into HIS broth and incubated at 30°C at 200 rpm for 2 h, the culture was then adjusted to an OD_600_ of 0.05, and 2 mL bacterial cells was added to each well, in which a submerged glass coverslip was present, in 24-well microplates. Following incubation of the microplates at 30°C at 100 rpm for 24 h, the coverslip was removed and rinsed once with PBS. Next, 10 μL 1× SYTO-9 (cell permeable, 485/535 nm; Thermo) was dropped onto the coverslip, and the coverslip was stained for 15 min. The biofilms were observed using an Olympus FV 1000 single-photon laser scanning microscope with a 20× lens objective.

For SEM analysis, the biofilm grown on a glass coverslip was obtained as mentioned above. The biofilm was washed once with 200 μL PBS and fixed with 100 μL 2.5% (vol/vol) glutaraldehyde. After dehydration and coating with copper, the biofilms were observed by SEM (JSM-6390LV; JEOL).

For SERS analysis, the biofilm was grown on a glass coverslip as described above. Next, the spectra of macromolecules in biofilms were scanned at 400 to 1,800 cm^−1^ using a SERS system (LabRam HR Evolution; Horiba, France) equipped with a 50× lens objective. LabSpec6 software was used to analyze the Raman data, and Origin2018 was then used for image processing.

Pictures shown for CLSM, SEM, and SERS are from one of three independent experiments.

### RT-PCR and RT-qPCR assays.

V. parahaemolyticus strains grown overnight were diluted 1:100 into HIS broth, ∼10-mL cultures were incubated in borosilicate glass tubes at 30°C at 100 rpm for 24 h, and a 2-mL culture was then centrifuged to extract the RNAs using TRIzol (Invitrogen). Residual DNA was removed using DNase (Turbo DNase; Ambion) according to the manufacturer’s instructions. For RT-PCR assays, a Superscript one-step RT-PCR system (Invitrogen) was used according to the manufacturer’s instructions. The pictures shown are from one of three independent experiments.

For qRT-PCR assays, RNAs were collected as mentioned above. TB Green fast qPCR (quantitative PCR) mix (TaKaRa) was used according to the manufacturer’s instructions. *rpoA* was used as an internal control for normalization. The results were analyzed using the comparative threshold cycle method. The data were the averages from three independent experiments.

### Bioinformatic analysis.

Sequences of ScvC, ScvL, and ScvO were used as queries to run the PSI-BLAST program (February 2020). Phylogenetic analysis was performed using MEGA X (30). RAST was used to annotate genomes (31). IS Finder (https://www-is.biotoul.fr/blast.php) was used to identify insertion sequences. SMART (http://smart.embl-heidelberg.de/) was used to identify and annotate protein domains.

### Statistical analysis.

Statistical analysis was performed using Prism (version 5.0; GraphPad Software) with unpaired two-tailed Student’s *t* test (*, *P < *0.05; **, *P < *0.01; ***, *P < *0.001; ns, not significant [*P > *0.05]).

### Data availability.

Genomic sequences of bacterial species used in this study were downloaded from the NCBI database. The annotated genomes are available at https://figshare.com/articles/journal_contribution/Genomes_of_scv_loci_positive_bacteria_species_annotated_using_RAST_/17871989.
