# Supplementary material for: A Polysaccharide Biosynthesis Locus in Vibrio parahaemolyticus Important for Biofilm Formation Has Homologs Widely Distributed in Aquatic Bacteria Mainly from Gammaproteobacteria
Source: mSystems. 2022 Mar 1;7(2):e01226-21. doi: 10.1128/msystems.01226-21 (PMC8941931; doi:10.1128/msystems.01226-21)
Supplement: TABLE S1 [file msystems.01226-21-st001.docx]

**Table S1. Putative functions of Scv proteins.**

| **Protein** | **Predicted function** | **Similarity in *A. fischeri*** | **Homologue in *A. fischeri*** |
| --- | --- | --- | --- |
| ScvA | Anti-anti-sigma factor | 58% | SypA |
| ScvB | Outer membrane protein | 56% | SypB |
| ScvC | Periplasmic protein involved in polysaccharide export | 64% | SypC |
| ScvD | ATPase involved in chromosome partitioning | 41% | SypD |
| ScvE | Response regulator | 67% | SypG |
| ScvF | Glycosyltransferase | 43% | SypH |
| ScvG | Glycosyltransferase | 41% | SypI |
| ScvH | Glycosyltransferase | 38% | SypJ |
| ScvI | Oligosaccharide translocase | 33% | SypK |
| ScvJ | Polysaccharide ligase | 58% | SypL |
| ScvK | Glycosyltransferase | 50% | SypN |
| ScvL | Polysaccharide export protein | 56% | SypO |
| ScvM | Glycosyltransferase | 51% | SypP |
| ScvN | Glycosyltransferase | 50% | SypQ |
| ScvO | Sugar transferase | 64% | SypR |
