# Supplementary figures and images for: A Polysaccharide Biosynthesis Locus in Vibrio parahaemolyticus Important for Biofilm Formation Has Homologs Widely Distributed in Aquatic Bacteria Mainly from Gammaproteobacteria
Source: mSystems. 2022 Mar 1;7(2):e01226-21. doi: 10.1128/msystems.01226-21 (PMC8941931; doi:10.1128/msystems.01226-21)

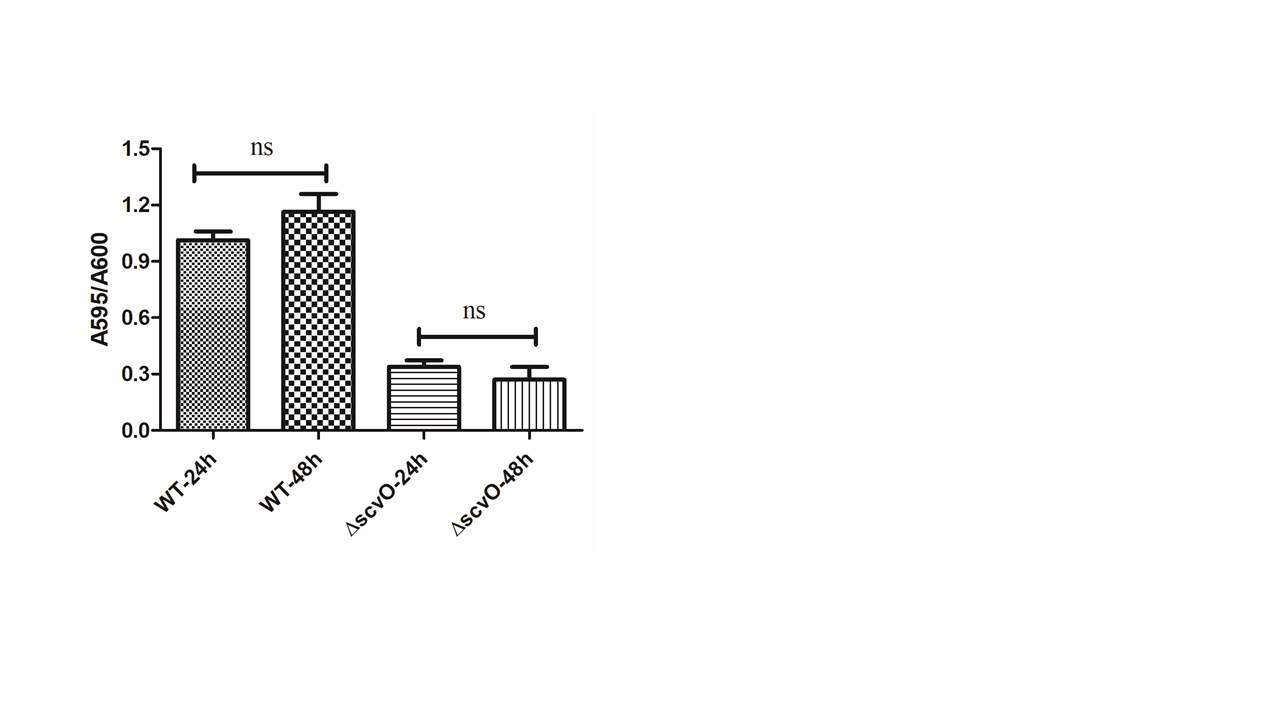

Supplement: FIG S1 [file msystems.01226-21-sf001.tif]

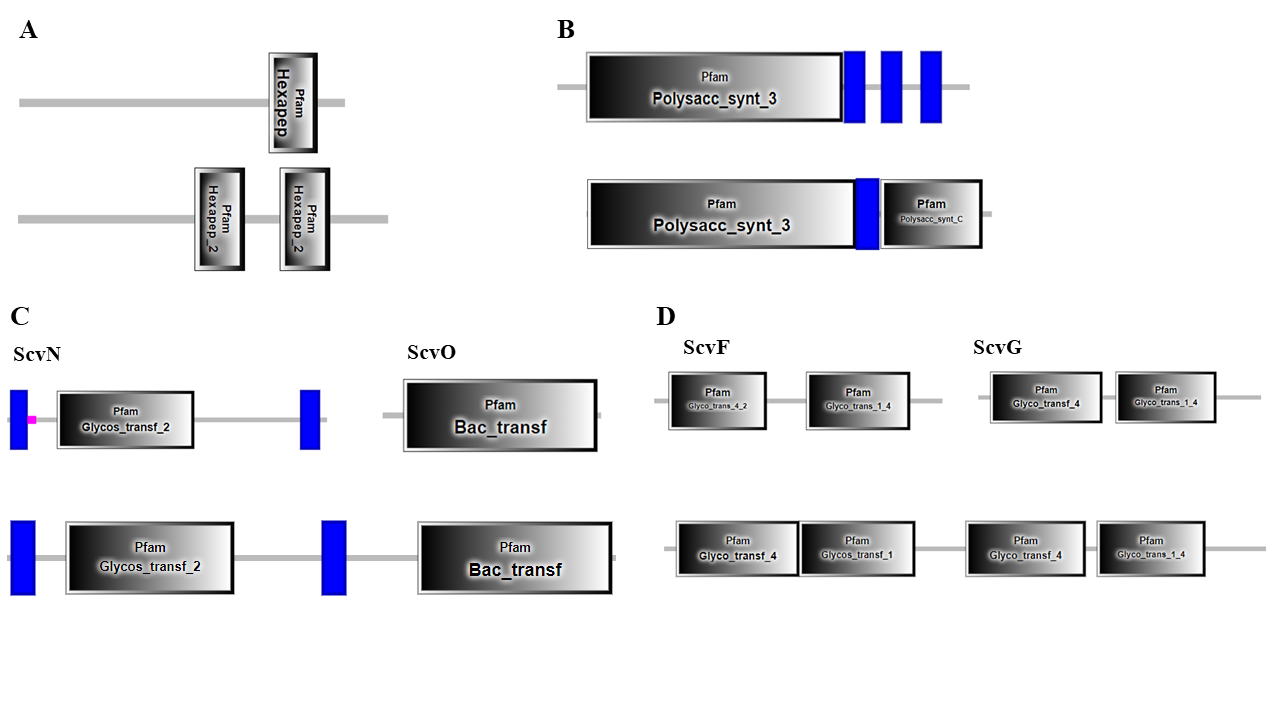

Supplement: FIG S2 [file msystems.01226-21-sf002.tif]
