# Supplementary material for: A Polysaccharide Biosynthesis Locus in Vibrio parahaemolyticus Important for Biofilm Formation Has Homologs Widely Distributed in Aquatic Bacteria Mainly from Gammaproteobacteria
Source: mSystems. 2022 Mar 1;7(2):e01226-21. doi: 10.1128/msystems.01226-21 (PMC8941931; doi:10.1128/msystems.01226-21)
Supplement: TABLE S2 [file msystems.01226-21-st002.docx]

**Table S2. Strains and plasmid used in this study.**

| Strain or plasmid | Description | Reference or source |
| --- | --- | --- |
| *E.coli* |  |  |
| 17-1 λ*pir* | *thi* *pro* *hsdR* *hsdM*^+^ *recA* PR-4-2-Tc::Mu-Km::Tn*7* λ*pir* | Lab collection |
| *V. parahaemolyticus* |  |  |
| RIMD 2210633 | pandemic strain, *tdh*^+^, *t3ss1*^+^, *t3ss2*^+^ | Makino *et al*., 2003 |
| Δ*cpsA* | *vpa1403* gene deletion mutant | This study |
| Δ*scvA* | *vp1476* gene deletion mutant | This study |
| Δ*scvJ* | *vp1464* gene deletion mutant | This study |
| Δ*scvO* | *vp1458* gene deletion mutant | This study |
| Δ*scvE* | *vp1469* gene deletion mutant | This study |
| Δ*scvO*Δ*cpsA* | *vp1458* and *vpa1403* deletion mutant | This study |
| Δ*aphA* | *vp2762* gene deletion mutant | This study |
| Δ*scvE*:pScvE | Δ*scvE* complemented with *scvE* gene | This study |
| Δ*scvE*:pScvE^D53A^ | Δ*scvE* complemented with *sypE-*D53A | This study |
| Plasmids |  |  |
| pDM4 | Cm^r^; suicide vector with an R6K origin and *sacBR* genes from *Bacillus subtilis* | Lab collection |
| pMMB207 | Cm^r^; RSF1010 derivative, *IncQlacI*^q^Cm^r^P*tacoriT* | Lab collection |

**References**

Makino K, Oshima K, Kurokawa K, Yokoyama K, Uda T, Tagomori K, Iijima Y, Najima M, Nakano M, Yamashita A, Kubota Y, Kimura S, Yasunaga T, Honda T, Shinagawa H, Hattori M, Iida T. 2003. Genome sequence of *Vibrio parahaemolyticus*: a pathogenic mechanism distinct from that of *V cholerae*. *Lancet* **361**: 743-749.
