# Supplementary material for: A Polysaccharide Biosynthesis Locus in Vibrio parahaemolyticus Important for Biofilm Formation Has Homologs Widely Distributed in Aquatic Bacteria Mainly from Gammaproteobacteria
Source: mSystems. 2022 Mar 1;7(2):e01226-21. doi: 10.1128/msystems.01226-21 (PMC8941931; doi:10.1128/msystems.01226-21)
Supplement: TABLE S3 [file msystems.01226-21-st003.docx]

**Table S3. Primers used in this study.**

| **Knockout** |
| --- |
| *cpsA* |
| 1F CCGCTCGAGCTGCTCACCGTTGTCACCTA |
| 1R TTGTTTTCATCATCGCCGCCGACCTAGTTTCCCTTCTAGC |
| 2F GCTAGAAGGGAAACTAGGTCGGCGGCGATGATGAAAACAA |
| 2R GAAGATCTCTTGGGTTTTCCCCGATATT |
| *aphA* |
| 1F GCTCTAGATAGGCGCAGGTGAAGCTATT |
| 1R TGTCTCTAAGTAAAAGGAAAGTCTTCAATCCAAATGGTCA |
| 2F TGACCATTTGGATTGAAGACTTTCCTTTTACTTAGAGACA |
| 2R CCGCTCGAGGGAGTGCCACAGGGAGAGTA |
| *scvE* |
| 1F GCTCTAGAGCAGTTCTGCACTGGCATTA |
| 1R TTGTTCGAGATTAACTTCATAGTCCTATCCCAACTTAATC |
| 2F GATTAAGTTGGGATAGGACTATGAAGTTAATCTCGAACAA |
| 2R ACATGCATGCCGCGACAAAGTTATTGAGCA |
| *scvA* |
| 1F GAAGATCTTCACGTTAATGGCACGGTTA |
| 1R CTCCGTTTAAACCGGTATTTGACCCTATCTCCATTTAGGT |
| 2F ACCTAAATGGAGATAGGGTCAAATACCGGTTTAAACGGAG |
| 2R CGAGCTCAGCAATGCGATTTTGCTTCT |
| *scvQ* |
| 1F GCTCTAGAGCCAAAAGCTAACGACGAAG |
| 1R AGCAAAATAGACTCGAAGCAGATGCTTTCCTTATTGGTTG |
| 2F CAACCAATAAGGAAAGCATCTGCTTCGAGTCTATTTTGCT |
| 2R CCGCTCGAGCGGACTCTGGTTCATTGGTT |
| *scvJ* |
| 1F CCGCTCGAGGCTGCTTCAAGTGATGGTGA |
| 1R TTGTTGTCAAAATGAAAGGACGCAATCACGACGGTGATCG |
| 2F CGATCACCGTCGTGATTGCGTCCTTTCATTTTGACAACAA |
| 2R CGAGCTCCGGGTTACACGCTTTGATTT |
| **Complementary** |
| scvE-F CGAGCTCTAAGGAGGTAGGATAATAatgagaccaagggtgttac |
| scvE-R CGGGATCCtcacttgtcatcgtcatccttgtaatcGgattgttcgagattaacttc |
| **RT-PCR** |
| 1 F AAATCAACCTCCAGCACGTC |
| 1 R AGCAATGCGATTTTGCTTCT |
| 2 F AACGAGCCGAGCAAGTTAAA |
| 2 R TCGTTATCTGCGGCATGTAG |
| 3 F CGAGCTGGGGATACGATTTA |
| 3 R CGTCTTTTAGGCTCGGAGTG |
| 4 F cactccgagcctaaaagacg |
| 4 R tagatgcagtgccacagagg |
| 5 F AAAGAAAACGCAGGCAAGAA |
| 5 R ATGCCTTTTGCCAGCTCTAA |
| 6 F ACGCCAAACGATCGTAGAAC |
| 6 R AGGTGCAAGCCAAGAGAAAA |
| 7 F CGTAGGAGAAGCGGCATTAG |
| 7 R AGCTCTGCATTTGCGATTTT |
| 8 F GAGTCACCGTGGATGAAGGT |
| 8 R CAAAGCAAAGCTGACCAACA |
| 9 F CTCGGTATTTTTGGCGTGTT |
| 9 R CGTGGATACGGAAAAAGGAA |
| 10 F CGTGGCCAATCTACATCCTT |
| 10 R GAGTGTGGAGCCGGTATTGT |
| 11 F AACAAAGCTGGGAAGCAAGA |
| 11 R TCAATCAGCCCTTGCTCTTT |
| 12 F GGTCTAGCCGCCATACTTGA |
| 12 R TCGCTAAGGATTGGGATTTG |
| 13 F CGCGCTTACATTCTTCAACA |
| 13 R AGCAATCCACTGCTCTTCGT |
| 14 F CGCCGTATTTGATGATTGTG |
| 14 R CTTGATGGCAAGAGCAATGA |
| **qRT-PCR** |
| rpoA-F TCGCCGCATTCTTCTATCTT |
| rpoA-R TCAGCGTTGTCATCCGTTAG |
| scvO-F gttcattgctcttgccatca |
| scvO-R cgagggccaatcatagacat |
| cpsA-F taccgttttggcctatttgc |
| cpsA-R atttgatcccagcgagaatg |
| scvD-F ACCTCTCCGTTGCTCAGTGT |
| scvD-R CGTCTTTTAGGCTCGGAGTG |
| **Site-directed mutagenesis** |
| D53A-F CCTCAACTGATCATTTTGGCCTTAAAACTTCCAGACATG |
| D53A-R CATGTCTGGAAGTTTTAAGGCCAAAATGATCAGTTGAGG |
